# Health care availability, quality, and unmet need: a comparison of transgender and cisgender residents of Ontario, Canada

**DOI:** 10.1186/s12913-017-2226-z

**Published:** 2017-04-18

**Authors:** Rachel Giblon, Greta R. Bauer

**Affiliations:** Department of Epidemiology & Biostatistics, Schulich School of Medicine & Dentistry, The University of Western Ontario, K201 Kresge Building, London, ON N6H 2B1 Canada

**Keywords:** Transgender, Health services, Disparities, Inequalities

## Abstract

**Background:**

Evidence suggests that transgender (trans) individuals in Canada are a medically underserved population; barriers range from lack of provider knowledge on trans issues to refusal of care. This paper provides the first formal estimation of health care inequalities between trans and cisgender individuals in Ontario, Canada.

**Methods:**

Weighted statistics from the Ontario-wide Trans PULSE Project (*n* = 433) were compared with age-standardized Ontario data from the Canadian Community Health Survey (*n* = 39,980) to produce standardized prevalence differences (SPDs). Analysis was also conducted separately for trans men and trans women, each compared to the age-standardized Ontario population.

**Results:**

An estimated 33.2% (26.4,40.9) of trans Ontarians reported a past-year unmet health care need in excess of the 10.7% expected based on the age-standardized Ontario population. Inequality was greatest comparing trans with cisgender men (SPD = 34.4% (23.0, 46.1). While trans Ontarians evaluated health care availability in Ontario similarly to the broader population, they were significantly more likely to evaluate availability in their community as fair or poor.

**Conclusions:**

Trans Ontarians experience inequalities in perception and reported experiences of health care access, with 43.9% reporting a past-year unmet health care need.

## Background

The term transgender (trans) is an umbrella term used that encompasses those whose gender identities do not match their birth-assigned sex. Trans persons may hold a broad spectrum of gender identities, such as trans, genderqueer, cross-dresser, transsexual, transitioned, Two-spirit and androgynous; however, not all individuals with these identities actually identify as trans. Furthermore, researchers too often have used either a narrow definition of trans (e.g., those attending gender clinics) or failed to disaggregate the concerns of trans people with those of sexual minorities like lesbian, gay and bisexual persons.

Data on the health-related experiences of trans persons in Canada can be collected using survey measures that identify trans respondents. Yet, current population health surveys (e.g., the Canadian Community Health Survey or CCHS) and census forms only allow respondents to identify as male or female; non-binary, trans, and intersex populations are not represented [1], precluding the ability of researchers to accurately assess if and where health inequalities exist between trans and cisgender (non-trans) populations, or to estimate the number of trans individuals living in Canada. Thus, prior studies on trans health have relied heavily on convenience samples, producing results that may not be representative of the broader trans population and also provide no point of comparison to the cisgender population. Using best available evidence – a conservative frequency estimate of 0.6% [2], extrapolated to 2016 Canadian census counts [3] – there are approximately 200,000 trans individuals’ aged 18 and older living in Canada, of which about 77,000 are located in Ontario.

In Canada, each of the 13 provinces and territories is individually responsible for providing essential medical services via a universal, publicly funded, health insurance program. However, despite this, previous research in Canada on the health of trans individuals has revealed this population to be medically underserved in both primary and specialist care settings; barriers included lack of providers knowledgeable about trans issues [4–6], denial of health care altogether [6–8] and/or refusal to approve hormone therapy and/or gender-affirming surgeries [9]. Consequently, health care avoidance remains high amongst trans persons: an estimated 21% (95% CI =14,25) of trans Ontarians have avoided going to the emergency department when emergency care was needed, explicitly because of concerns related to accessing emergency department services as a trans individual [6]. Of those who had accessed the emergency department while expressing a gender different from their birth-assigned sex, 52% (95% CI = 34, 72) had experienced negative treatment due to being trans, ranging from insulting or demeaning language to outright refusal of care [6]. Similar findings were seen in a nation-wide survey of Canadian trans youth: 47% of older youth (14–18 years) and 33% of younger youth (19–25 years) reported that they had not received needed health care in the past 12 months with 61% indicating that this was due to being “afraid of what the doctor would say or do” [10]. Moreover, while 83.1% (95% CI = 77, 89) of trans Ontarians had a regular family doctor, approximately half indicated they were not comfortable discussing trans issues with their regular health care provider [8].

Although these studies indicate unmet health care need is a serious issue among trans communities in Canada, their results have not been accurately compared to data from the general population. Our study is the first of its kind in that it estimates inequalities in both health care use and perceptions of quality and availability by providing valid, standardized comparisons between trans and (presumed) cisgender residents of Ontario, using two population-based samples. Thus, our study makes an important contribution to the literature in that it provides a concrete assessment of the magnitude of trans health inequalities.

## Methods

### Data sets

This study used Ontario data from two sources collected in 2009–2010: the Canadian Community Health Survey (CCHS) and the Trans PULSE survey. The CCHS is an extensive national cross-sectional telephone survey developed by Statistics Canada [11]. Currently, data collection is ongoing, with complete weighted cycles released every two years. For the 2009/2010 cycle, data were collected from 39,980 respondents aged 16 and older living in Ontario, representing approximately 10.5 million Ontarians [12]. Data on health, health care, and health determinants for persons living in private dwellings in Canada were collected via a complex, multi-stage, stratified cluster design; excluded from this survey were persons living on Aboriginal reserves and crown lands, in institutions, or in some remote areas, as well as full-time members of the Canadian Armed Forces and those who are unstably housed or homeless [11, 12]. Given that approximately 99.4% of the population will be cisgender [2], in this analysis the CCHS data will stand in for the experiences of (presumed) cisgender Ontarians.

Trans PULSE was a community-based research project designed to identify and address issues surrounding health, healthcare access and social services use within trans communities in Ontario [13]. Respondent-driven sampling (RDS) methodology, appropriate for sampling hidden populations [14], was utilized to gather survey data from 433 trans individuals aged 16 and older living in Ontario during 2009 and 2010 [15]. Participants had the option to complete the survey online, on paper, or via telephone. After completing the survey, they were provided with three tracked coupons that could be used to recruit additional participants, and recruitment continued to a maximum of 10 waves beyond the initial 38 participants. Trans PULSE survey items on health care access were drawn from existing CCHS items, to allow for comparisons between the two data sets.

### Measures

Demographic information was collected for both trans and the general Ontario population. Participants in Trans PULSE were coded as trans men if they were on the transmasculine spectrum, meaning that they reported they were assigned female at birth, but now identified as men, trans men, genderqueer or other gender identities. Similarly, trans women were coded as those who were assigned male at birth and indicated a range of female or non-binary identities.

To assess perceptions of health care, respondents in both data sets were asked a series of four questions: 1) “Overall, how would you rate the availability of health care services in Ontario?” 2) “Overall, how would you rate the quality of the health care services available in Ontario?” 3) “Overall, how would you rate the availability of health care services in your community?” 4) “Overall, how would you rate the quality of the health care services in your community?” Response options were: excellent, good, fair, and poor. To evaluate unmet health care need, respondents were asked to answer yes or no to the following question: “During the past 12 months, was there ever a time when you felt that you needed health care but didn’t receive it?”

### Analysis

#### Trans PULSE prevalence estimates

RDS is a form of non-random sampling; well-connected respondents are more likely to be recruited than others. To correct for this, prevalence estimates were weighted based on probability of recruitment using RDS I methods [16, 17], in RDS Analysis Tool version 7.1.4 (RDSAT) software. This method accounts for transition probabilities (different likelihoods of certain groups recruiting others) and network size (knowing different numbers of eligible participants), and in doing so controls for homophily bias, the tendency of participants to know and recruit those who are more like themselves. 95% confidence intervals (CIs) were calculated from the distribution of 15,000 replicate bootstrap samples, using a specialized RDS bootstrapping algorithm [16, 17]. All prevalences were estimated for the full trans population, and then stratified by gender spectrum (trans men or trans women).

#### CCHS prevalence estimates

To account for the CCHS’s complex sampling strategy, a SAS macro program called BOOTVAR (developed by Statistics Canada [18]) was used to construct 95% CIs using CCHS-specific bootstrap weights, with 500 replicates. Bootstrapping methodology was not employed for the prevalence estimates themselves, which were computed within BOOTVAR by applying final person-level sampling weights to the total sample.

As differences in age and gender can independently influence key social determinants of health like education and income, we compared these for trans and Ontario populations. While the ratio of trans men to trans women in Ontario was not significantly different than the ratio of men to women in the Ontario population for all age groups, the trans community was, on average, younger: 33.2% (95% CI = 25,43) of trans Ontarians were aged 16–24 compared to only 14.4% (95% CI = 14,15) of the general Ontario population (data not shown). Therefore, all CCHS prevalence estimates for health care variables were age-adjusted using direct standardization to represent expected values among the (younger) trans population, and separately among trans men and trans women. To perform standardization, a secondary SAS macro (STD_MACRO) was employed concurrently with the BOOTVAR program. Using this program, weighted variable rates were calculated for each age stratum: 16–24, 25–34, 35–44, 45–54, 55–64, and 65+; this process was done using both total population person-level weights for point estimates, and the provided set of 500 bootstrap weights. Modelling the Ontario trans population (Trans PULSE) as our standard population, CCHS stratum-specific rates (overall and bootstrap) were then multiplied by the weight of each age group in the Ontario trans population to generate age-standardized prevalence estimates; the simple variance of these estimates across the 500 bootstrap samples was used to produce corresponding 95% CIs. These age-standardized prevalences for health access variables represent what would be expected for the cisgender Ontario population if it had the same age distribution as the trans population.

Age distribution also differed significantly for trans people by gender spectrum, with trans men being younger than trans women. Thus, two additional sets of prevalence estimates were generated using the same methodology as outlined above, standardized separately to the population age distributions of trans men and trans women. These age-standardized prevalences represent what would be expected of the Ontario population if it had the same age distribution as the population of trans men and (separately) trans women.

#### Standardized prevalence differences

Effect measures were reported as standardized prevalence differences (SPD’s) with 95% CI’s. SPDs were calculated by subtracting the age-adjusted prevalence estimates derived from CCHS data from Trans PULSE prevalence estimates. Thus, they represent the excess prevalence (if positive) or the reduced prevalence (if negative) among trans population versus what would be expected. For example, an SPD of 0.20 would indicate that 20% of trans Ontarians experienced an outcome that they would likely not have experienced if they were cisgender. Confidence intervals for differences between rates were computed using methods of variance estimates recovery (MOVER) [19]; this method utilizes the separate confidence limits of prevalences from each sample to construct a confidence interval for their difference. All SPDs and 95% CI MOVER calculations were preformed using a Microsoft Excel [20] spreadsheet.

## Results

Trans population demographics are displayed in Table 1. There were an approximately equal number of trans men and trans women. Trans men were, on average, younger than their trans women counterparts: 43.1% (34.6, 63.5) of trans men were aged 16–24 compared to only 23.9% (12.8, 47.0) of trans women.

**Table 1:** Weighted frequencies for demographics in the Ontario trans population

	All trans people (*n* = 433)		Trans men^a^ [1] (*n* = 205)		Trans women^b^ (*n* = 227)	
	%	95% CI	%	95% CI	%	95% CI
Gender spectrum						
Trans men	52.8	(44.8, 62.0)	--		--	
Trans women	46.9	(37.8, 55.0)	--		--	
Age						
16–24	33.2	(25.2, 43.1)	43.1	(34.6, 63.5)	23.9	(12.8, 47.0)
25–34	29.1	(22.4, 37.0)	34.1	(21.1, 43.6)	35.0	(20.3, 49.5)
35–44	16.4	(10.9, 22.4)	14.6	(4.9, 22.0)	16.9	(6.6, 27.9)
45–54	12.5	(6.9, 18.5)	8.1	(1.6, 13.4)	11.2	(2.1, 17.1)
55–64	6.3	(2.3, 9.8)	0	(**---**)	7.2	(0.7, 12.6)
65+	2.5	(0.6, 5.0)	0	(**---**)	5.6	(1.4, 16.5)
Ethno-racial identities^c^						
White	87.8	(82.4, 92.6)	79.6	(69.2, 88.7)	92.6	(88.9, 99.2)
Aboriginal	6.0	(2.9, 9.6)	7.4	(2.2, 15.9)	6.1	(1.0, 7.7)
Asian	7.0	(3.5, 11.5)	12.3	(4.6, 25.7)	4.2	(0.9, 10.0)
Latin American	3.5	(0.8, 7.0)	7.2	(1.9, 18.0)	0.0	(0.0, 0.0)
Black	2.9	(0.8, 5.8)	4.6	(0.6, 7.5)	1.0	(0.0, 1.3)
Middle Eastern	3.7	(1.1, 7.0)	7.0	(1.3, 13.1)	0.6	(0.0, 6.6)
Other	3.8	(0.9, 7.1)	5.9	(0.6, 16.2)	0.6	(0.0, 5.0)
Aboriginal identity						
Non-Aboriginal	94.4	(91.2, 97.3)	95.4	(88.4, 98.9)	93.3	(92.3, 98.6)
First Nations, Métis or Inuit	5.6	(2.7, 8.8)	4.6	(1.1, 11.6)	6.7	(1.4, 7.7)
Immigration history						
Immigrant	15.6	(9.9, 21.7)	22.1	(10.3, 34.5)	12.0	(4.9, 22.6)
Non-immigrant	81.8	(75.5, 88.0)	77.9	(65.5, 89.7)	82.2	(70.0, 90.7)
Other	2.5	(0.5, 5.4)	0.0	(**---**)	5.8	(0.7, 14.8)
Marital status						
Never married	61.0	(52.5, 69.0)	66.8	(55.4, 80.9)	62.0	(47.5, 78.6)
Separated	7.8	(4.1, 12.7)	3.0	(0.4, 11.1)	11.1	(3.7, 24.9)
Divorced	7.3	(3.5, 12.2)	3.9	(0.4, 6.4)	8.5	(0.4, 16.4)
Widowed	0.2	(0.0, 1.0)	0.6	(0.0, 3.2)	0.0	(0.0, 0.1)
Living common-law	9.3	(5.3, 14.3)	12.3	(4.0, 18.3)	3.6	(0.8, 16.3)
Married	14.4	(8.6, 19.9)	13.4	(4.9, 22.5)	14.7	(3.5, 17.5)
Region of province						
Eastern Ontario	14.9	(7.4, 24.5)	19.4	(7.8, 40.9)	11.8	(0.5, 28.4)
Central Ontario	16.8	(10.7, 25.0)	7.1	(1.0, 23.5)	26.4	(13.7, 41.6)
Metropolitan Toronto	32.7	(21.5, 42.1)	39.8	(25.9, 64.5)	24.7	(11.4, 37.6)
Southwestern Ontario	27.3	(16.7, 38.7)	29.7	(7.3, 32.5)	23.3	(7.3, 47.4)
Northern Ontario	8.4	(3.0, 16.3)	4.0	(0.2, 6.8)	13.8	(1.4, 28.4)
Education						
High school not completed	12.5	(8.1, 18.7)	13.3	(4.1, 15.7)	12.4	(4.6, 21.3)
High school graduate	16.2	(10.8, 21.5)	18.2	(11.9, 30.6)	13.2	(4.4, 21.7)
Some postsecondary	28.2	(22.3, 35.6)	28.9	(22.3, 45.1)	31.3	(20.2, 45.7)
College or university degree	35.6	(27.8, 42.7)	32.5	(18.7, 40.7)	37.7	(27.0, 53.3)
Graduate or prof degree	7.6	(3.6, 11.6)	7.2	(1.5, 15.2)	5.5	(0.8, 8.1)
Current student						
Yes, full-time	23.3	(16.8, 31.0)	26.9	(19.2, 42.9)	19.0	(7.4, 32.0)
Yes, Part-time	5.7	(3.6, 9.3)	7.7	(3.4, 14.6)	2.1	(0.2, 5.7)
No	71.1	(62.4, 77.3)	65.4	(48.2, 73.2)	78.9	(65.6, 90.4)
Household income						
Less than $15, 000	29.4	(19.8, 37.4)	24.8	(14.7, 44.1)	31.1	(14.2, 44.4)
$15, 000 to $29, 999	17.3	(11.4, 24.6)	16.7	(6.8, 23.9)	16.5	(8.2, 32.4)
$30, 000 to $49, 999	23.3	(16.4, 31.9)	34.7	(21.1, 49.5)	10.8	(3.3, 21.3)
$50, 000 to $79, 999	12.7	(7.4, 19.1)	10.4	(4.1, 17.4)	14.4	(3.6, 21.7)
$80, 000 +	17.2	(10.7, 25.1)	13.4	(4.1, 22.4)	27.2	(14.0, 48.5)
Personal Income						
Less than $15, 000	48.2	(40.2, 57.9)	50.1	(38.7, 66.0)	49.4	(35.4, 67.2)
$15, 000 to $29, 999	21.0	(14.8, 28.8)	20.6	(10.8, 31.5)	18.3	(6.4, 29.3)
$30, 000 to $49, 999	17.1	(10.3, 22.5)	21.7	(9.3, 29.7)	8.5	(3.5, 20.2)
$50, 000 to $79, 999	7.1	(3.0, 10.9)	3.2	(0.9, 8.0)	13.0	(3.3, 22.1)
$80, 000 +	6.6	(2.7, 12.4)	4.5	(0.1, 12.7)	11.0	(1.4, 23.5)
Employment status						
Full-time job	42.5	(32.9, 53.5)	37.0	(24.8, 53.2)	45.8	(28.9, 66.8)
More than one part-time job	12.8	(7.6, 19.2)	13.9	(6.4, 27.8)	13.2	(6.6, 29.3)
One part-time job	24.1	(16.5, 34.3)	34.4	(18.0, 44.9)	11.7	(2.2, 14.8)
Retired	1.7	(0.0, 6.6)	0.4	(0.0, 2.6)	5.8	(0.0, 20.7)
Student (not working)	4.5	(1.1, 9.9)	5.6	(0.2, 20.6)	4.4	(0.0, 2.8)
Unemployed	10.3	(3.7, 15.0)	6.4	(0.0, 11.5)	15.4	(2.5, 29.9)
Permanently unable to work	4.1	(0.3, 7.1)	2.4	(0.0, 3.3)	3.6	(0.0, 10.0)

^a^Respondents were categorized as trans men if they were female at birth and now identified as boy or man, FTM, trans boy or man, feel like a boy sometimes, she-male, two-spirit, intersex, crossdresser, genderqueer, or bigender

^b^Respondents were categorized as trans women if they were male at birth and now identified as girl or women, MTF, trans girl or women, feel like a girl sometimes, she-male, two-spirit, intersex, crossdresser, genderqueer, or bigender

^c^Check all that apply; percentages may not add up to 100%

### Standardization to the overall trans population

Comparisons of health-care variables for trans population and the age-adjusted Ontario population are presented in Table 2. During 2009–2010, past-year unmet health care need was reported by 43.9% of trans Ontarians versus 10.7% expected based on the age-adjusted population. This represents an excess prevalence of 33.2% (26.4, 40.9), meaning that an estimated 1 in 3 trans Ontarians experienced an unmet health need in the last year that would not be expected were they not trans. Moreover, a higher percentage of trans participants felt that health care quality (SPD = 11.3% (5.1, 16.4)) and availability (SPD = 7.4% (1.4, 14.1)) in their community were poor during this time period. Additionally, although trans persons assessed health care availability in Ontario similarly to expectations based on cisgender Ontarians, an excess of 7.1% (1.9, 13.6) of trans respondents indicated a poor quality of health care in Ontario.

**Table 2:** Weighted frequencies for health care assessment and unmet need in Ontario trans population, compared with age-adjusted frequencies for Ontario (Canadian Community Health Survey)

	Trans people *N* = 433		Ontarians, age-standardized to trans population *N* = 39,980		Standardized Prevalence Difference	
	%	95% CI	%	95% CI	%	95% CI
Healthcare availability in Ontario						
Excellent	13.0	(8.1, 18.5)	14.9	(14.2, 15.7)	−1.9	(−6.9, 3.6)
Good	48.4	(40.8, 55.8)	51.5	(50.5, 52.6)	−3.1	(−10.8, 4.4)
Fair	27.5	(20.7, 34.5)	25.1	(24.1, 26.0)	2.4	(−4.5, 9.5)
Poor	11.1	(6.4, 17.2)	8.5	(8.0, 9.0)	2.6	(−2.1, 8.7)
Healthcare quality in Ontario						
Excellent	12.7	(7.6, 17.9)	17.3	(16.5, 18.1)	−4.6	(−9.8, 0.7)
Good	50.7	(42.1, 58.5)	56.9	(55.8, 57.9)	−6.2	(−14.9, 1.7)
Fair	24.4	(18.3, 32.2)	20.9	(20.1, 21.7)	3.5	(−2.7, 11.3)
Poor	12.1	(6.9, 18.6)	5.0	(4.5, 5.4)	7.1	(1.9, 13.6)* [2]
Community healthcare availability						
Excellent	12.3	(7.9, 17.4)	15.7	(14.9, 16.4)	−3.4	(−7.9, 1.8)
Good	37.5	(30.2, 44.8)	49.1	(48.1, 50.1)	−11.6	(−19.0, −4.2)*
Fair	31.5	(24.1, 38.9)	24.0	(23.1, 24.8)	7.5	(0.1, 15.0)*
Poor	18.7	(12.7, 25.7)	11.3	(10.7, 11.9)	7.4	(1.4, 14.4)*
Community healthcare quality						
Excellent	11.7	(7.0, 14.5)	16.7	(15.9, 17.5)	−5.0	(−9.8, −2.1)*
Good	48.5	(37.1, 52.0)	57.1	(56.1, 58.1)	−8.6	(−20.0, −5.0)*
Fair	22.3	(15.7, 27.8)	20.3	(19.2, 20.9)	2.0	(−4.6, 7.6)
Poor	17.5	(11.3, 22.6)	6.2	(5.7, 6.6)	11.3	(5.1, 16.4)*
Needed healthcare in the past year but did not receive it						
Yes	43.9	(37.2, 51.5)	10.7	(9.7, 11.6)	33.2	(26.4, 40.9)*
No	56.1	(48.5, 62.8)	89.2	(88.3, 90.1)	−33.1	(−40.8, −26.3)*

*p < 0.05

### Standardization to the trans male population

Standardizing the Ontario population separately to the age distribution of trans men for comparison with this group (Table 3) revealed that a significantly larger percentage of trans men had not received needed health care in the past year, compared to age-standardized expectations for cisgender men (SPD = 34.4% (23.0, 46.1)) and cisgender women (SPD = 29.2% (17.2, 40.9). Interestingly, slightly fewer trans men found the availability of healthcare in Ontario to be poor, compared to expectations based on both cisgender men (SPD = −3.1% (−6.9, −2.1)) and women (SPD = −4.4% (−8.4, −3.2), and there were no differences in assessment of healthcare quality in Ontario or healthcare availability in their community. However, more trans men felt healthcare quality in their community was poor than was expected based on age-standardized estimates for cisgender men or women.

**Table 3:** Weighted frequencies for health care assessment and unmet need in Ontario trans men, compared with frequencies for Ontario men and women (Canadian Community Health Survey) age-adjusted to the population of trans men

	Trans men *N* = 227		Ontario men *N* = 17,869		Standardized Prevalence Difference		Ontario women *N* = 22,111		Standardized Prevalence Difference	
	%	(95% CI)	%	(95% CI)	%	(95% CI)	%	(95% CI)	%	(95% CI)
Healthcare availability in Ontario										
Excellent	16.4	(10.3, 27.8)	16.1	(14.7, 17.4)	0.3	(− 5.9, 11.8)	14.2	(13.0, 15.4)	2.2	(−4.0, 13.7)
Good	50.4	(40.8, 65.7)	51.7	(49.8, 53.6)	−1.3	(−11.1, 14.1)	53.5	(52.0, 55.1)	−3.1	(−12.8, 12.3)
Fair	29.4	(16.9, 36.9)	25.1	(23.4, 26.8)	4.3	(−8.3, 12.0)	23.8	(22.6, 25.1)	5.6	(−7.0, 13.2)
Poor	3.9	(0.2, 4.5)	7.0	(6.2, 7.9)	−3.1	(−6.9, −2.1)*	8.3	(7.5, 9.2)	−4.4	(−8.2, −3.4)* [3]
Healthcare quality in Ontario										
Excellent	15.8	(8.6, 26.7)	19.1	(17.7, 20.5)	−3.3	(−10.6, 7.7)	15.9	(14.6, 17.2)	−0.1	(−7.4, 10.9)
Good	47.9	(35.7, 63.1)	56.7	(54.9, 58.6)	−8.8	(−21.1, 6.5)	57.6	(56.0, 59.2)	−9.7	(−22.0, 5.6)
Fair	25.3	(14.0, 34.2)	19.9	(18.4, 21.4)	5.4	(−6.0, 14.4)	21.3	(20.1, 22.6)	4.0	(−7.4, 13.0)
Poor	11.0	(2.9, 19.4)	4.2	(3.5, 4.9)	6.8	(−1.3, 15.2)	5.1	(4.3, 5.9)	5.9	(−2.2, 14.3)
Community healthcare availability										
Excellent	16.3	(8.5, 25.6)	16.6	(15.2, 17.9)	−0.3	(−8.2, 9.1)	15.1	(14.0, 16.2)	1.2	(−6.7, 10.6)
Good	37.6	(26.3, 53.0)	50.6	(48.8, 52.4)	−13.0	(−24.4, 2.5)	48.5	(46.8, 50.2)	−10.9	(−22.3, 4.6)
Fair	27.8	(15.9, 38.0)	23.2	(21.7, 24.7)	4.6	(−7.4, 14.9)	24.5	(23.1, 25.9)	3.3	(−8.7, 13.6)
Poor	18.3	(8.5, 28.5)	9.5	(8.6, 10.5)	8.8	(−1.1, 19.0)	11.8	(10.8, 12.8)	6.5	(−3.4, 16.7)
Community healthcare quality										
Excellent	16.9	(9.3, 26.6)	18.0	(16.5, 19.4)	−1.1	(−8.8, 8.7)	15.3	(14.2, 16.5)	1.6	(−6.1, 11.4)
Good	41.7	(33.8, 60.1)	57.4	(55.7, 59.1)	−15.7	(−23.8, 2.8)	57.2	(55.6, 58.9)	−15.5	(−23.6, 3.0)
Fair	18.8	(8.3, 26.0)	19.2	(1.7, 20.6)	−0.4	(−11.0, 18.5)	21.2	(20.9, 22.6)	−2.4	(−13.0, 4.8)
Poor	22.6	(10.1, 30.0)	5.4	(4.6, 6.2)	17.2	(4.7, 24.6)*	6.1	(5.5, 6.8)	16.5	(4.0, 23.9)*
Needed healthcare in the past year but did not receive it										
Yes	42.3	(31.0, 53.9)	7.9	(6.7, 9.1)	34.4	(23.0, 46.1)*	13.1	(11.6, 14.6)	29.2	(17.8, 40.9)*
No	57.7	(46.1, 69.0)	91.8	(90.5, 93.1)	−34.1	(−45.8, −22.7)*	86.8	(85.3, 88.2)	−29.1	(−40.8, −17.7)*

*p < 0.05

### Standardization to trans female population

Finally, in comparison with expectations based on both cisgender men and women, in 2009–2010 a greater proportion of trans women felt health care availability in their community was only fair, but they did not differ from cisgender expectations in their perception of overall health care quality, or availability in Ontario (Table 4). Nevertheless, as with trans men, a much higher proportion of trans women reported having not received needed health care in the past year, with standardized prevalence differences as high as 28.1% (19.8, 48.4) over cisgender men and 23.5% (15.2, 43.8) over cisgender women.

**Table 4:** Weighted frequencies for health care assessment and unmet need in Ontario trans women, compared with frequencies for Ontario men and women (Canadian Community Health Survey) age-adjusted to the population of trans women

	Trans women *N* = 205		Ontario men *N* = 17,869		Standardized Prevalence Difference		Ontario women *N* = 22,111		Standardized Prevalence Difference	
	%	(95% CI)	%	(95% CI)	%	(95% CI)	%	(95% CI)	%	(95% CI)
Healthcare availability in Ontario										
Excellent	14.2	(7.0, 30.8)	15.7	(14.6, 16.8)	−1.5	(−8.8, 15.1)	14.0	(13.0, 15.0)	0.2	(−7.1, 16.8)
Good	42.9	(33.3, 56.9)	50.2	(48.7, 51.7)	−7.3	(−17.0, 6.8)	51.3	(50.0, 52.5)	−8.4	(−18.1, 5.7)
Fair	27.0	(12.9, 34.3)	25.5	(24.1, 26.9)	1.5	(−12.7, 8.9)	25.3	(24.2, 26.4)	1.7	(−12.4, 9.1)
Poor	15.9	(4.5, 26.1)	8.4	(7.6, 9.2)	7.5	(−3.9, 17.7)	9.2	(8.5, 10.0)	6.7	(−4.7, 16.9)
Healthcare quality in Ontario										
Excellent	12.4	(3.9, 24.3)	19.0	(17.9, 20.1)	−6.6	(−15.2, 5.4)	15.5	(14.5, 16.5)	−3.1	(−11.7, 8.8)
Good	51.7	(42.2, 68.5)	56.2	(54.7, 57.7)	−4.5	(−14.1, 12.4)	56.8	(55.5, 58.1)	−5.1	(−14.7, 11.8)
Fair	26.1	(13.5, 38.5)	19.8	(18.6, 21.1)	6.3	(−6.4, 18.8)	22.1	(21.0, 23.2)	4.0	(−8.6, 16.4)
Poor	9.8	(0.7, 15.5)	4.8	(4.1, 5.5)	5.0	(−4.1, 10.7)	5.4	(4.8, 6.0)	4.4	(−4.7, 10.1)
Community healthcare availability										
Excellent	9.4	(3.2, 23.3)	16.5	(15.4, 17.6)	−7.1	(−13.4, 6.8)	14.7	(13.7, 15.6)	−5.3	(−11.6, 8.6)
Good	36.8	(23.7, 47.4)	49.2	(47.7, 50.7)	−12.4	(−25.6, −1.7)*	47.9	(46.5, 49.3)	−11.1	(−36.5, −0.4)* [4]
Fair	34.9	(25.2, 53.1)	23.5	(22.2, 24.8)	11.4	(1.6, 29.6)*	24.8	(23.6, 26.1)	10.1	(0.3, 28.3)*
Poor	18.9	(6.3, 23.3)	10.6	(9.8, 11.4)	8.3	(−4.3, 12.8)	12.4	(11.6, 13.3)	6.5	(−6.1, 11.0)
Community healthcare quality										
Excellent	8.3	(3.6, 20.5)	18.0	(16.8, 19.2)	−9.7	(−14.6, 2.6)	15.3	(14.3, 16.3)	−7.0	(−11.8, 5.2)
Good	54.5	(39.3, 69.7)	56.6	(55.1, 58.1)	−2.1	(−17.4, 13.2)	56.7	(55.4, 58.0)	−2.2	(−17.5, 13.1)
Fair	26.1	(13.6, 39.3)	19.1	(17.9, 20.3)	7.0	(−5.6, 20.3)	21.2	(20.1, 22.4)	4.9	(−7.7, 18.1)
Poor	11.0	(3.1, 15.9)	6.1	(5.3, 6.9)	4.9	(−3.0, 9.9)	6.6	(6.0, 7.2)	4.4	(−3.5, 9.3)
Needed healthcare in the past year but did not receive it										
Yes	36.4	(28.2, 56.7)	8.3	(7.2, 9.5)	28.1	(19.8, 48.4)*	12.9	(11.6, 14.2)	23.5	(15.2, 43.8)*
No	63.6	(43.3, 71.8)	91.3	(90.1, 92.5)	−27.7	(−48.0, −19.4)*	86.8	(85.5, 88.1)	−23.2	(−43.5, −14.9)*

*p < 0.05

## Discussion

Consistent with existing literature on health and health services in trans communities [7, 21, 22], a large proportion of trans individuals reported having needed healthcare in the past year that they did not receive. This proportion was significantly higher than would be expected for cisgender Ontarians, with one-third of trans Ontarians reporting a past-year unmet health need in excess of expectations. Trans individuals were more likely to rate the quality of health care in their community as poor, despite having a positive assessment of health care availability in Ontario. Hence, it appears that equal recognition of the availability of health care in their province (though not always in their community) does not translate into equal access.

There are multiple possibilities for excess prevalence of unmet need, including that trans individuals may have higher need for medical services, and/or be less likely to access care. Existing evidence does not suggest a higher need for emergency services [6], but among the approximately three-quarters of trans people who require hormones or surgeries, there may be excess need for primary or specialist care at particular periods of life based on trans-specific healthcare needs. Moreover, some studies point to an increased prevalence of HIV (in trans women), depression, tobacco use, and alcohol addiction within trans communities [1, 10, 22, 23] Research certainly supports the existence of barriers to healthcare for trans persons, including an absence of formal education of health care providers on trans issues (an issue identified by both physicians [4] and trans community members [5]), and high frequencies of harassment and discriminatory practices experienced by trans individuals in health care settings [6, 8]. An analysis of access to emergency services found that 21% of trans people in Ontario had avoided going to the emergency department in a medical crisis specifically because they were trans [6].

Gender-stratified analysis showed that standardized prevalence differences for unmet health care need were large for both trans men and women, in comparisons of each to expectations for both cisgender men and women. While an approximately equal proportion of trans men and women rated healthcare availability in their community to be poor, twice as many trans men felt that the quality of the healthcare in their community was also poor. This trend was reversed for perceived healthcare availability and quality in Ontario, with substantially more trans women than men rating healthcare availability in their province as poor, and roughly equal numbers feeling health care quality was poor. Thus, while trans men perceive better health care availability than trans women, they also suffer from greater levels of unmet health care need compared to cisgender Ontarians, possibly stemming from feelings of poorer healthcare quality. Previous studies have shown transphobia and discrimination are more pronounced in the workplace for trans men – it’s possible that this trend also extends to health care [24, 25]. Nevertheless, the percentage of trans men and women reporting having had unmet health care needs in the past year were roughly equivalent (42.3% of trans men vs. 36.4% of trans women), and overall, there were no statistically significant differences between trans men and women in health and perceptions of health care. These results are congruent with a previous Trans PULSE analysis on risk factors for not having a regular family practitioner, which found no differences in the bivariate association between gender identity (quantified as male-to-female or female-to male) and not having a family practitioner [Scheim A, Zong X, Giblon R, Bauer G: Disparities in access to family physicians among transgender people in Ontario, Canada, submitted.].

In it important to note that our results may not accurately represent current differences. Educational and policy changes implemented in the time since data collection for this study was completed in 2010 have been designed both specifically to improve health care access, and to eliminate barriers to social participation more generally. The Ministry of Health and Long-term Care has funded a program (Trans Health Connection) to provide trans-related clinical care and cultural competency training for health care providers in cities and towns across the province. This is a promising approach given the prior finding that trans patient perception of a lack of physician knowledge of trans issues has been associated with patient discomfort with their physician [8]. In 2012, the Human Rights Tribunal of Ontario ruled sex reassignment surgery was no longer a requirement for changing one’s sex/gender on provincial birth certificates [26]; surgical requirements were then quickly eliminated for change of sex/gender designation on provincial health cards, and the display of designations on these cards is now being phased out entirely. In 2014, the Ontario Human Rights Commission added gender identity and expression as explicitly prohibited grounds of discrimination [27]; and in 2015, parliament introduced Bill C-16, which if passed into legislation, would amend the Canadian Human Rights Act and the Criminal Code to include gender identity and gender expression [28].

More recent data are not available from which to identify to what extent inequalities observed in this study might be reduced; however, plans are currently underway to shape an expanded project aimed at collecting data on trans individuals nationwide. However, even expanded data collection within trans communities will not allow for assessment of inequalities between trans and cisgender populations. For this reason, identification of trans participants within large population surveys such as the CCHS is needed to more accurately assess a greater range of inequalities, and to track changes over time.

## Conclusions

In conclusion, this study highlights the disparity in unmet health care need that existed between trans Ontarians and their cisgender counterparts, despite similarities in perceptions of health care between these two populations. Further research into why trans people in Ontario are either not accessing or unable to access primary and specialty care services, despite Canada’s ‘universal health insurance system’, is needed.

## Abbreviations

CCHS: Canadian community health survey
CI: Confidence interval
HIV: Human immunodeficiency virus
RDS: Respondent-driven sampling
RDSAT: Respondent-driven sampling analysis tool
SPD: Standardized prevalence difference
